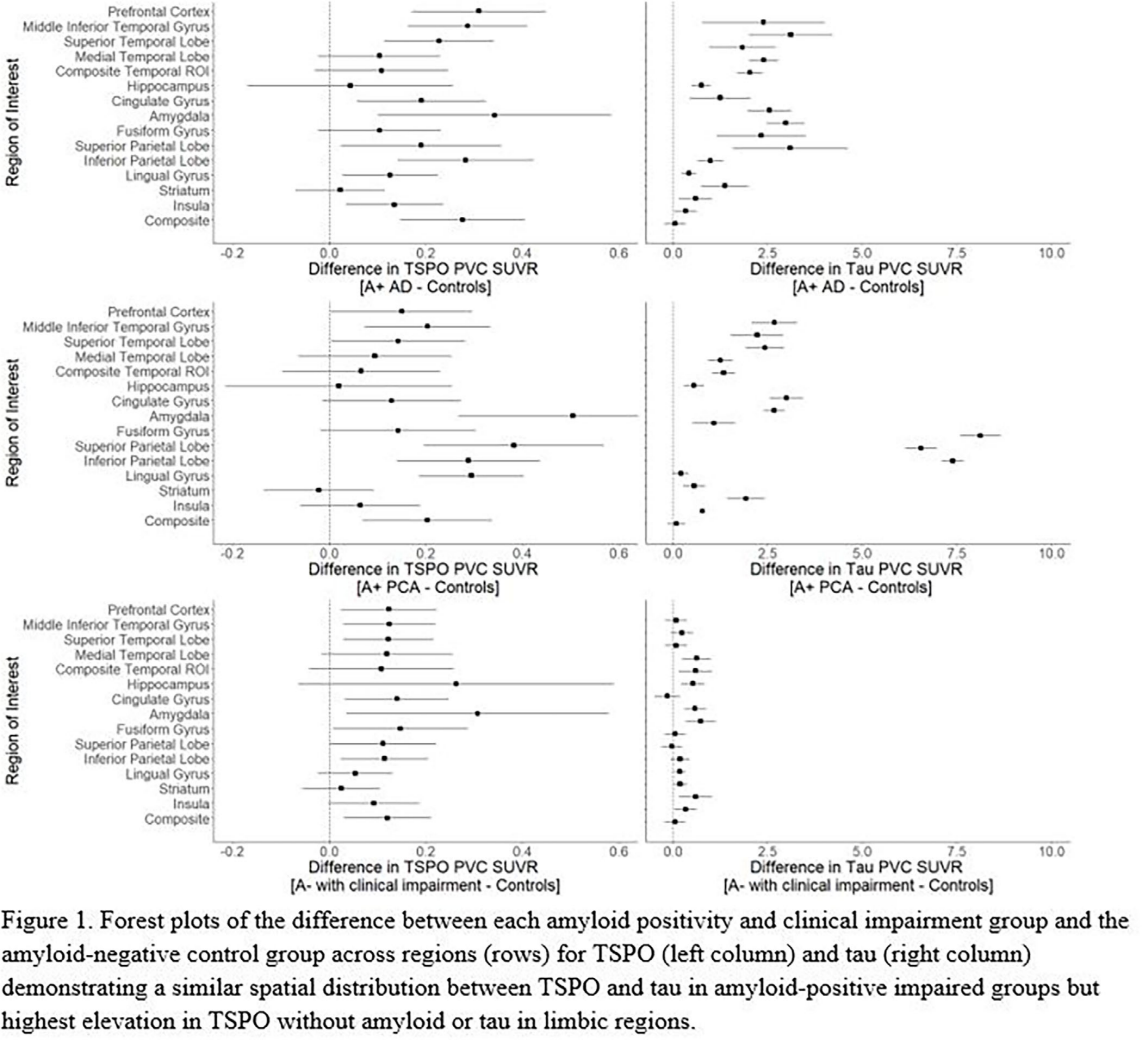# Microglia density measured by TSPO PET across amyloid positivity and clinical variants

**DOI:** 10.1002/alz.095057

**Published:** 2025-01-09

**Authors:** Hannah M. Houlihan, Aubrey S. Johnson, Anna C. Smith, Diana S. Guzmán, Amarachukwu Okafor, Lauren B. Heuer, Daniel Talmasov, Ndubisi Chikwem, Dina S. Dass, James M. Noble, William C. Kreisl, Scott A. Small, Patrick J. Lao

**Affiliations:** ^1^ Columbia University Irving Medical Center, New York, NY USA

## Abstract

**Background:**

We hypothesized that TSPO PET, which measures microglia density, would be elevated in the presence of amyloid and impairment across different clinical variants in a pattern that follows their characteristic tau distribution.

**Method:**

Participants (n = 17 amyloid‐negative control, 3 amyloid‐positive AD, 2 amyloid‐positive PCA, 6 amyloid‐negative with impairment (1 aMCI, 1 MCI, 3 AD, 1 LATE); age = 69±7, 43% women) from the Longitudinal Imaging of Microglial Activation in Different Clinical Variants of Alzheimer’s Disease study underwent amyloid PET (Florbetaben), tau PET (MK6240), and TSPO PET (ER176). Amyloid positivity was determined by visual read. Clinical groups were determined at ADRC consensus. Partial volume corrected TSPO and tau SUVR was compared across amyloid positivity and clinical variants (amyloid‐negative controls as reference group).

**Result:**

The amyloid‐positive AD group had elevated TSPO in amygdala (0.34, p = 0.01), prefrontal cortex (0.31, p = 0.0004), middle inferior temporal gyrus (0.29, p = 0.0003), inferior parietal lobe (0.28, p = 0.001), superior temporal lobe (0.23, p = 0.001), cingulate gyrus (0.19, p = 0.01), superior parietal lobe (0.19, 0.04), insula (0.14, p = 0.02), and lingual gyrus (0.13, p = 0.02), while the amyloid‐positive PCA group had elevated TSPO in amygdala (0.50, p = 0.001), superior parietal lobe (0.38, p = 0.04), inferior parietal lobe (0.29, p = 0.001), middle inferior temporal gyrus (0.20, p = 0.0003), prefrontal cortex (0.15, p = 0.0004), superior temporal lobe (0.14, p = 0.001), cingulate gyrus (0.13, p = 0.01), and insula (0.06, p = 0.02). The impaired amyloid‐negative group had elevated TPSO in the amygdala (0.31, p = 0.04), fusiform gyrus (0.15, p = 0.05), cingulate gyrus (0.14, p = 0.02), middle inferior temporal gyrus (0.12, p = 0.02), superior temporal lobe (0.12, p = 0.02), prefrontal cortex (0.12, p = 0.02), and inferior parietal lobe (0.11, p = 0.02). These elevations in TSPO spatially coincide with elevations in tau burden (Figure 1).

**Conclusion:**

Microglia may respond to amyloid pathology and follow a spatial pattern similar to that of tau pathology for a given clinical variant. Interestingly, microglia were elevated to the greatest extent in key limbic regions for amyloid‐negative impaired participants. Non‐AD pathologies may be driving this limbic neuroinflammation or limbic neuroinflammation itself may be sufficient for clinical impairment in the context of low amyloid and tau burden.